# Antimicrobial resistance: A multifaceted problem with multipronged solutions

**DOI:** 10.1002/mbo3.945

**Published:** 2019-11-14

**Authors:** Teresa Gil‐Gil, Pablo Laborda, Fernando Sanz‐García, Sara Hernando‐Amado, Paula Blanco, José Luis Martínez

**Affiliations:** ^1^ Centro Nacional de Biotecnología CSIC Madrid Spain

**Keywords:** antibiotic resistance, One Health, Global Health, Infectious diseases

## Abstract

Infectious diseases still stand as a major cause of morbidity and mortality, and this problem can be worsened with the current antimicrobial resistance crisis. To tackle this crisis more studies analyzing the causes, routes, and reservoirs where antimicrobial resistance can emerge and expand, together with new antimicrobials and strategies for fighting antimicrobial resistance are needed. In the current special issue of MicrobiologyOpen, a set of articles dealing with the multiple faces of antimicrobial resistance are presented. These articles provide new information for understanding and addressing this problem.

## Graphical abstract

Infectious diseases still stand as a major cause of morbidity and mortality, and this problem can be worsened with the current antimicrobial resistance crisis. In order to tackle this crisis more studies analyzing the causes, routes, and reservoirs where antimicrobial resistance can emerge and expand, together with new antimicrobials and strategies for fighting antimicrobial resistance are needed. In the current special issue of MicrobiologyOpen, a set of articles dealing with the multiple faces of antimicrobial resistance are presented. These articles provide new information for understanding and addressing this problem.

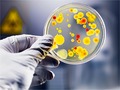

## 1 COMMENTARY

Antimicrobial resistance (AMR) is a relevant problem for human health as recognized by several independent agencies, governments, and international organizations, including the WHO and the UN among others (Govindaraj Vaithinathan & Vanitha, 2018). The problem is not restricted to human‐linked habitats, since different works have shown that other ecosystems, including animals, soil and water bodies contribute to the origin, spread and maintenance of AMR, hence being a One Health problem (Berendonk et al., 2015). Besides, AMR bacteria (AMRB) can expand through different geographical areas, which constitutes a Global Health problem (Hernando‐Amado, Coque, Baquero, & Martinez, 2019). Understanding the elements involved in the emergence of resistance as well as the ways of counteracting such resistance, requires then integrated approaches where the ecological and evolutionary aspects of antimicrobial resistance are taken into consideration (Baquero, Alvarez‐Ortega, & Martinez, 2009). In the current special issue on Antimicrobial Resistance, MicrobiologyOpen presents a set of articles covering different areas of AMR, from the study of reservoirs and vectors for its dissemination to the analysis of novel antimicrobial compounds with potential therapeutic use.

Besides being a relevant health problem, AMRB and AMR genes (AMRGs) released from human‐linked reservoirs constitute nowadays a pollution problem. Thus, knowing where and how they can be inactivated is a subject of interest (Martinez, 2012). This issue has been addressed by analyzing the levels and diversity of AMRGs in sandy beach environments compared with mangroves of South China, these last receiving sewage and aquaculture wastewater from coastal regions (Zhao et al., 2019). Notably, the authors found that the total abundance of AMRGs is lower in mangrove sediments. Despite the authors state that the properties of the sediment and the presence of mobile genetic elements are the most relevant elements in shaping the AMRGs, it is relevant to state that, as described by others (Forsberg et al., 2014), most analyzed genes are intrinsic AMRGs, indicating that the phylogenetic composition of the community should also be relevant. One important element for understanding the spread of AMR is the delimitation of the transmission routes. Wild birds, particularly migratory ones, can contribute to such dissemination, being a potential vector for AMR spread among geographically distinct areas (Bonnedahl & Jarhult, 2014). The analysis of fecal samples from short‐ and long‐distance migratory wild birds in Switzerland (Zurfluh et al., 2019) showed that 5.8% of the birds contained AMR *Escherichia coli* and several of them harbored clinically‐relevant extended‐spectrum beta‐lactamases as CTX‐M‐15, CTX‐M‐55, or CTX‐M‐65. Since several of these birds are present in both, urbanized and natural environments, they could potentially spread AMRBs within and between these environments.

One of the challenges to face AMR is the lack of quantitative models for analyzing its dissemination. A probabilistic model to estimate cross‐contamination and recontamination by methicillin‐resistant *Staphylococcus aureus* in retail meat via hands and cookware during a household barbecue is presented in (Plaza‐Rodriguez, Kaesbohrer, & Tenhagen, 2019). According to this model, the probability of one ARMB to be transferred from the contaminated raw chicken meat to the final serving is low. The application of this model to other potential transmission routes may help in quantifying the relevance of each one of them. These models can be fueled by the genomic analysis of recent opportunistic pathogens, particularly those with an environmental origin that can spread through human‐linked and environmental reservoirs. This is the case of *Elizabethkingia anophelis*, an emerging and frequently misdiagnosed opportunistic pathogen, whose AMR spectra and pathogenesis mechanisms are still unclear. The genome of a clinical isolate presenting resistance to 20 antibiotics was sequenced, and genes encoding different beta‐lactamases and efflux pumps, as well as several potential virulence factors, were found (Wang et al., 2019). The comparison of three clinical and two environmental *E. anopheles* strains showed that they present similar AMRGs and virulence determinants, further highlighting the role that natural ecosystems may have in the spread of AMRBs (Martinez, 2008). Even once the genomes of a pathogenic species have been sequenced, and the molecular basis for AMR predicted, fast, reliable, and cheap methods for their detection is needed. One example of this situation is the *Aeromonas* genus, in which the phenotypic identification of species causing human infections is not always easy. Proteomic identification by MALDI‐TOF MS, linked to classical susceptibility tests, emerges as a valuable tool, in terms of time‐efficiency and low‐costs, for solving this issue (Elbehiry et al., 2019). It is important to notice that fast detection and molecular epidemiological surveys of AMR require previous knowledge of the molecular basis of resistance (McArthur & Wright, 2015); and predictive approaches are of utmost relevance for this purpose (Martinez, Baquero, & Andersson, 2007). Two articles in this special issue make use of these methods to predict the emergence of resistance to two antimicrobials produced by lactic bacteria: nisin (Arii, Kawada‐Matsuo, Oogai, Noguchi, & Komatsuzawa, 2019), an antibacterial peptide used as a food preservative, and the bacteriocin plantaricin EF (Heeney, Yarov‐Yarovoy, & Marco, 2019). Mutations that lead to plantaricin resistance are located in the *corC* gene, which encodes a putative membrane‐bound magnesium/cobalt efflux protein, which is supposed to be the bacteriocin target. Thus, the mechanism of action of plantaricin EF could be linked to modifications in the bacterial metal homeostasis. *S. aureus*, on its hand, acquires resistance to nisin as a consequence of the selection of mutations in the two‐component system BraRS, which in turn leads to the increased expression of the VraDE efflux pump, also known to be involved in daptomycin and gallidermin resistance (Popella et al., 2016). These results highlight the fact that the acquisition of resistance to one antibiotic can modify the susceptibility to others, either reducing (cross‐resistance) or increasing (collateral sensitivity) bacterial susceptibility to them. While the analysis of cross‐resistance phenotypes gives clues for avoiding the use of some specific antibiotics when bacteria have acquired resistance to another one, the analysis of the collateral sensitivity networks can help to develop combined or cyclic therapeutic regimes (Podnecky et al., 2018; Sanz‐Garcia, Hernando‐Amado, & Martinez, 2018). Indeed, *Pseudomonas aeruginosa* mutants selected in the presence of carbapenems presented decreased susceptibility to doripenem, meropenem, and imipenem, while their susceptibility to aminoglycosides, fluoroquinolones, and noncarbapenem β‐lactams was higher than that of the parental clinical isolate from which these mutants derive (Harrison, Fowler, Abdalhamid, Selmecki, & Hanson, 2019). While alterations in the porin OprD, the entrance channel for carbapenems, were responsible for the increased resistance to these antibiotics, modifications in the lipopolysaccharide were likely the cause of aminoglycoside hypersensitivity. These evolutionary constraints imposed by collateral sensitivity could be exploited in the clinics to the rational design of treatments.

Most studies on AMR are based on the analysis of resistance to industrially produced antimicrobials used in therapy or for prophylactic purposes. Nevertheless, it is important to recall that the infected organisms can produce a set of antimicrobial compounds and knowing the bacterial mechanisms of resistance to such compounds is of utmost relevance for understanding infection. As shown by Desloges et al., (2019), the capacity of uropathogenic *E. coli* to resist being killed by antimicrobial peptides during urinary tract infections (UTIs) is mediated by the protease OmpT. Noteworthy, the presence of another *ompT‐*like gene (*arlC*) causes higher clinical severity in the UTIs. Therefore, the presence of this second AMRG should be taken into consideration for diagnostic and prognostic purposes in *E. coli* urinary infections.

One of the main causes of the increasing problem of AMR is the lack of novel antimicrobials or therapeutic approaches (Bush et al., 2011), a matter that is also explored in the current special issue of MicrobiologyOpen. To that purpose, different strategies could be implemented. The search of naturally‐produced compounds is exemplified by the article of Horta et al., (Horta et al., 2019), where the biodiversity of bacteria associated with the red seaweed *Asparagopsis armata*, together with the production of antimicrobial compounds by these microorganisms, is analyzed. Two of the investigated isolates produce potent anti–Gram‐positive antimicrobials, suggesting that the microbiota associated with *A. armata* can be a source of compounds with pharmacological interest. Genome mining of antimicrobial producers may also help in developing novel antimicrobials (Desloges et al., 2019). Using this approach, Nakaew et al., (Nakaew, Lumyong, Sloan, & Sungthong, 2019) showed that an antibiotic‐producing *Streptomyces* sp. isolate encodes in its genome, not only biosynthetic pathways of already known antimicrobials but also pathways predicted to be involved in the biosynthesis of novel antibiotics, which, as above stated, are urgently needed. Most antibiotics currently in use are of natural origin or derived from natural antimicrobials. However, purely synthetic antimicrobials as quinolones can also effectively inhibit bacterial pathogen growth. A synthetic approach was followed by Serafim et al., (Serafim et al., 2019) for synthesizing new 1,3‐bis(arloxy)propan‐2‐amines with biological activity against Gram‐positive pathogens such as *Streptococcus pyogenes*, *Enterococcus faecalis*, and *S. aureus*. By using docking approaches, the authors suggest that their compounds might bind to the cell division protein FtsZ. Although these predictions are far away to be confirmed, anti‐FtsZ antimicrobials should constitute a novel group of antibiotics with clear clinical value (Panda et al., 2016).

It is relevant to remind that the use of antimicrobials is not restricted to the treatment of human infections. They are also used in animal and crop production. Consequently, the use of new anti‐infective approaches for avoiding infections associated with these activities would reduce the global antimicrobial load, which will have a positive effect in reducing the AMR burden. One of these approaches can be combination therapy as described by Procopio et al (Procopio et al., 2019). In their study, the authors showed that the lectin CasuL is a bacteriostatic and antibiofilm agent against some Staphylococcal isolates obtained from animals with mastitis. Besides, it presents a synergistic activity when used in combination with antibiotics, supporting the need for conducting new studies to determine the feasibility of this approach in the treatment of caprine and bovine mastitis. A classical way for avoiding infections in crops is the development of transgenic plants with anti‐infective properties. Khademi et al., (Khademi, Nazarian‐Firouzabadi, Ismaili, & Shirzadian Khorramabad, 2019) developed transgenic tobacco plants which were able to produce dermaseptin B1, an antimicrobial peptide produced by *Phyllomedusa bicolor*. The purified recombinant peptide presented a strong antimicrobial activity, suggesting it can be useful as a novel compound for treating plants infections. Also, the results suggest that transgenic plants expressing dermaseptin B1 might be resistant to infection.

The diversity of the articles published in this special issue of MicrobiologyOpen supports that, as stated in the title, antibiotic resistance is a multifaceted problem that requires multipronged solutions.

## CONFLICT OF INTEREST

None declared.

## AUTHOR CONTRIBUTIONS

Writing‐original draft: TGG, PL, FSG, SHA, PB; Writing‐review and editing: TGG, PL, FSG, SHA, PB, JLM; Conceptualization: JLM; Resources: JLM; Supervision: JLM.

## ETHICAL APPROVAL

None required.

## Data Availability

Not applicable.
